# Personalized Treatment of Advanced Gastric Cancer Guided by the MiniPDX Model

**DOI:** 10.1155/2022/1987705

**Published:** 2022-01-27

**Authors:** Jianzheng Wang, Jinxi Huang, Hui Wang, Wei Yang, Qiwen Bai, Zhentao Yao, Qingli Li, Huifang Lv, Beibei Chen, Caiyun Nie, Weifeng Xu, Shuiping Tu, Hongle Li, Xiaobing Chen

**Affiliations:** ^1^Department of Medical Oncology, Affiliated Cancer Hospital of Zhengzhou University, Henan Cancer Hospital, Zhengzhou, Henan Province 450008, China; ^2^Department of General Surgery, Affiliated Cancer Hospital of Zhengzhou University, Henan Cancer Hospital, Zhengzhou, Henan Province 450008, China; ^3^Department of Endoscope Center, Affiliated Cancer Hospital of Zhengzhou University, Henan Cancer Hospital, Zhengzhou, Henan Province 450008, China; ^4^Department of Oncology, Renji Hospital, School of Medicine, Shanghai Jiaotong University, Shanghai 200127, China; ^5^Department of Molecular Pathology, Affiliated Cancer Hospital of Zhengzhou University, Henan Cancer Hospital, Zhengzhou, Henan Province 450008, China

## Abstract

**Background:**

The morbidity and mortality of gastric cancer are high in China. There are challenges to develop precise and individualized drug regimens for patients with gastric cancer after a standard treatment. Choosing the most appropriate anticancer drug after a patient developing drug resistance is very important to improve the patient's prognosis. MiniPDX has been widely used as a new and reliable preclinical research model to predict the sensitivity of anticancer drugs.

**Methods:**

The OncoVee® MiniPDX system developed by Shanghai LIDE Biotech Co., Ltd. was used to establish the MiniPDX models using specimens of patients with gastric cancer. The cancer tissues were biopsied under endoscopy, and then, the tumor cell suspension was prepared for a drug sensitivity test by subcutaneously implanting into Balb/c-nude mice. The selected optimal regimen obtained from the MiniPDX assay was used to treat patients with drug-resistant gastric cancer.

**Results:**

We successfully established an individualized and sensitive drug screening system for four patients from January 2021 to July 2021. MiniPDX models identified potentially effective drugs for these four patients, with partial remission in two of the patients after treatment and disease progression in the remaining of two patients. Severe side effects from chemotherapy or targeted therapy were not observed in all patients.

**Conclusion:**

Establishing a personalized drug screening system for patients with drug-resistant gastric cancer can guide the selection of clinical drugs, improve the clinical benefit of patients, and avoid ineffective treatments. It can be an effective supplement for treatment options.

## 1. Introduction

Gastric cancer is one of the most common malignancies worldwide and the fourth leading cause of cancer-related death [1]. The disease generally carries a dismal prognosis due to its advanced stage at initial diagnosis. The median survival rate is less than 12 months for patients at the advanced stage [2]. At present, the first-line treatment of metastatic gastric cancer is based on conventional chemotherapy, and there is an urgent need to explore a more effective treatment plan [3]. Except for the positive results of trastuzumab in HER2-positive advanced gastric cancer, other clinical studies on targeted drugs for the treatment of advanced gastric cancer ended in failure [4–6]. In recent years, immune checkpoint inhibitors have emerged for advanced gastric cancer with their unique mechanism of action. For HER2-negative gastric cancer, the two major clinical studies of CheckMate649 and Attraction04 have established the clinical utility of nivolumab for the first-line treatment [7, 8]. For HER2-positive gastric cancer, the practice-changing findings of the KEYNOTE-811 trial led the US FDA to grant accelerated approval of pembrolizumab in combination with trastuzumab and fluoropyrimidine and platinum-based chemotherapy as first-line therapy for patients with HER2-positive advanced gastroesophageal junction adenocarcinoma [9]. Therefore, in general, we have standard treatment options for the first-line treatment of advanced gastric cancer.

However, after the first-line or second-line treatment for patients with advanced gastric cancer, how to choose a personalized regimen with accuracy and effectiveness for each patient is currently a great challenge. Although there are many recommendations in the National Comprehensive Cancer Network (NCCN) guidelines or Chinese Society of Clinical Oncology (CSCO) guidelines, there is no final conclusion on which option is the most effective one [3, 10]. Although next-generation sequencing (NGS) and other genetic tests can be performed after disease progression, many patients cannot find suitable targets or suitable drugs from the test results. Therefore, it is essential to select effective drugs and develop a personalized treatment regimen.

In recent years, more and more researchers have tried to screen anticancer drugs in vitro by simulating tumor growth in vivo. Patient-derived xenograft (PDX) has emerged in this context and has achieved certain clinical results. It is currently the most representative animal model with human tumor genetic information [11–13]. The accuracy of PDX samples on drug efficacy and resistance rates can reach 90% based on a previous study [14]. Although the PDX drug sensitivity test has obvious advantages, it takes about half a year to go through the traditional PDX drug sensitivity test (the process of modeling, passage, amplification, and efficacy analysis) [12]. The survival time of patients with advanced gastric cancer is short, and the disease progression is rapid [15–17]. Due to the long construction time, the traditional PDX model cannot quickly reflect the drug sensitivity of patients and cannot meet the clinical needs. The latest rapid, human-derived xenograft tumor drug sensitivity detection technology (MiniPDX) solves this problem. These models generate drug sensitivity test results within 7–10 days [18]. Clinical studies have shown that MiniPDX can help improve the prognosis of patients with gallbladder cancer [19].

Therefore, in this study, MiniPDX was used to test the specimens of gastric cancer patients who had undergone pretreatment and select the best anticancer drugs for the subsequent treatment. Based on the MiniPDX assay results, we chose the most effective tumor-inhibiting regimen to evaluate whether it can effectively inhibit tumor growth in patients.

## 2. Materials and Methods

### 2.1. Collection of Patient Information

We collected a range of baseline information from each patient, including age, gender, medical history, tumor staging, pathological types, Lauren's classification, HER2 status, imaging examination results, and metastatic sites (Table 1).

### 2.2. Tissue Specimen Acquisition

We performed painless gastroscopy for tissue biopsy in patients with gastric cancer who have progressed after the first-line or second-line treatment under general anesthesia. In addition to routine pathological examination, we harvested enough tissue specimens for quality control before the establishment of MiniPDX models.

### 2.3. MiniPDX Model Establishment

**Figure 1:**
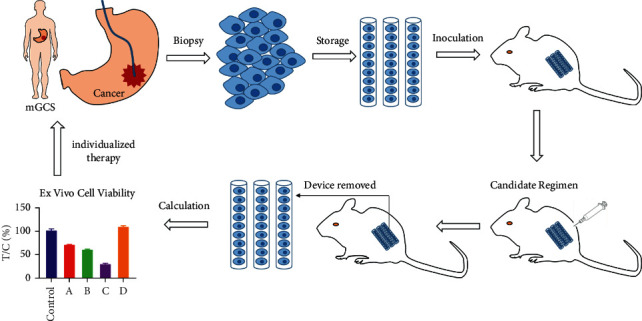
General schema of MiniPDX models of gastric cancer.

MiniPDX assay was carried out using the OncoVee® MiniPDX kit (LIDE Biotech Co., Ltd., Shanghai, China) (Figure 1). Gastric cancer samples were harvested and then washed with Hanks' balanced salt solution (HBSS) to remove nontumor and necrotic tumor tissues in a biosafety cabinet. Subsequently, the tumor tissues were digested with collagenase at 37°C for 1-2 h. Gastric cancer cells were collected followed by removal of blood cells and fibroblasts. Then, gastric cancer cell suspension was transferred to the HBSS washed OncoVee® capsules. Capsules were implanted subcutaneously via a small skin incision with three capsules per mouse (5-week-old nu/nu mouse), two mice per drug regimen [18]. One day after tumor cell inoculation, tumor-bearing mice were randomly allocated to vehicle group and drug treatment group. We chose 4 to 6 chemotherapy regimens for MiniPDX drug sensitivity tests according to the following principles: frequently used second-line and late-line chemotherapeutic plans and targeted plans for gastric cancer (such as docetaxel, irinotecan, nab-paclitaxel, S-1, and apatinib) were commonly considered; potentially effective molecular-targeted drugs such as pyrotinib and fruquintinib were also selected. Drugs that might cause severe side effects, as indicated by medical history and genetic testing results, were avoided. Drug sensitivity tests were carried out using MiniPDX models, as described later. Finally, we formulated a personalized therapeutic regimen for each patient according to the drug sensitivity test results.

### 2.4. Drug Sensitivity Test

Mice-bearing MiniPDX capsules were treated with single drugs or combination of drugs as detailed in Table 2 for 7 days. Thereafter, the implanted capsules were removed, and tumor cell proliferation was evaluated using the CellTiter Glo Luminescent Cell Viability Assay Kit, as instructed by the manufacturer. Luminescence was measured in terms of relative luminance unit (RLU) using a spectrophotometer. Relative proliferation rate (RPR) was calculated using the following equation: (mean RLU of the treatment group on day 7−mean RLU on day 0)/(mean RLU of the vehicle group on day 7−mean RLU on day 0) × 100%. Each experiment was conducted in sextuplicate, and the mean values were reported. A positive drug response was considered if RPR ≤55%, and a negative drug response was considered if RPR >55% [20].

### 2.5. The Prediction of Side Effects

The potential side effects of each protocol were investigated by determining the weight loss of mice in the MiniPDX system. Weight loss was recorded as RCBW% (rate of change in body weight) and was calculated as follows: RCBW% = (BWi − BW0)/BW0*∗*100%, in which BWi represented the body weight of the mouse on day 1 and BW0 represented the body weight of the mouse when the MiniPDX model began. Each regimen was tested in two mice, and final RCBW% was calculated as the average number of the two repeats; 15% was chosen as a cutoff point to predict whether we would consider avoiding this regimen due to the possibility of severe side effects [21].

### 2.6. Statistical Analysis

Statistical data and graphics were analyzed using GraphPad Prism 8. Data are presented as mean ± standard deviation (SD). Statistical significance was assessed by Student's *t* test, with *P* < 0.05 indicating significance.

## 3. Results

### 3.1. Basic Clinical Information

From January 2021 to July 2021, we constructed and tested the MiniPDX models in four patients with advanced gastric cancer. Before constructing the mouse models, we conducted in-depth communication with patients and their families, and each patient signed an informed consent form. The four patients with advanced gastric cancer have progressed after previous treatment, and there was no precise and effective plan to guide their subsequent treatment. To avoid ineffective attempts, we took biopsies of primary gastric cancer in these four patients under endoscopy and then tested the drug sensitivity by establishing the MiniPDX models (Table 1**)**. Patient #1 suffered from HER2-positive gastric cancer with liver metastases. Sintilimab combined with XELOX (L-OHP combined with capecitabine) was used as the first-line treatment. After treatment, the primary gastric cancer progressed, but the liver metastases shrank. Patient #2 had HER2-positive gastric cancer with liver metastasis. The first-line treatment was SOX (L-OHP + S-1) regimen for four cycles, followed by trastuzumab combined with SOX regimen for three cycles. Then, trastuzumab combined with S-1 was used for maintenance therapy. During the treatment, both primary gastric cancer and liver metastases have progressed. Patient #3 also had HER2-positive gastric cancer with liver metastases. The first-line treatment was trastuzumab combined with XELOX for 6 cycles. The primary gastric cancer progressed, and the liver metastases shrank. Patient #4 was diagnosed with diffuse gastric cancer with pelvic metastasis, and the pathological type was signet ring cell carcinoma. The first-line treatment was 3 cycles of camrelizumab, apatinib, and SOX. The primary gastric cancer and the pelvic metastases were significantly enlarged. Biopsies of all primary gastric tumors and metastases were performed in these four patients. However, no cancer cells were found in the liver metastases of patient #1 and patient #3, which might be related to the necrotic lesion after chemotherapy or targeted therapy. MiniPDX models failed to be established from the liver metastases of patient #2 and the pelvic metastases of patient #4 because tissues were too little to be punctured. In the end, the MiniPDX models were successfully established from the biopsy tissues of these four patients with primary gastric cancer lesions.

### 3.2. MiniPDX Drug Sensitivity Results

**Figure 2:**
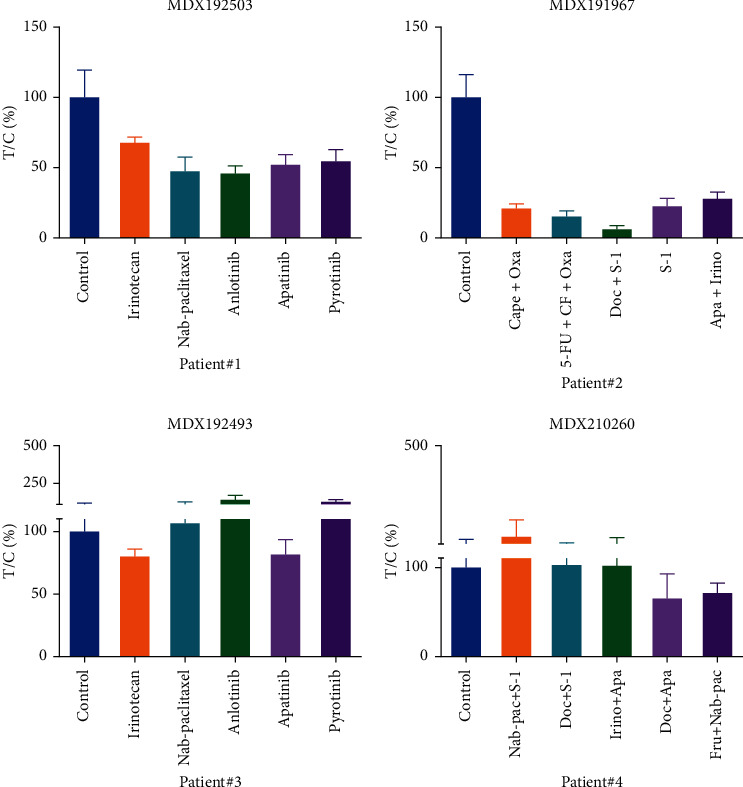
Drug selection test results for the MiniPDX models based on the four patients. Tumor cell growth (T/C% = treatment group proliferation rate/control group proliferation rate%) was calculated using the formula: (mean RLU of the treatment group on day 7-mean RLU on day 0)/(mean RLU of the vehicle group on day 7-mean RLU on day 0). Abbreviations for chemotherapy: Nab-pac (nab-paclitaxel); Cape (capecitabine); Oxa (oxaliplatin); Irino (irinotecan); Apa (apatinib); Doc (docetaxel); Fru (fruquintinib); 5-FU (5-fluorouracil); CF (calcium folinate).

A regimen was defined as “sensitive” when the proliferation rate of the tumor cells was under 55% compared to the control group [21]. The subsequent choice of the chemotherapeutic regimen was guided by the drug sensitivity test results. According to the test results of MiniPDX and the CSCO guidelines, we selected the most suitable regimen for treatment for each patient. Patient #1 received nab-paclitaxel as second-line treatment. Although the MiniPDX results showed that the inhibitory effect of anlotinib was slightly more potent than that of nab-paclitaxel, there is more evidence-based medicine for albumin paclitaxel as second-line treatment [22]. Patient #2 was treated with docetaxel combined with S-1 as second-line treatment because it has the most potent tumor inhibitory effect among the five chemotherapy regimens selected. It was challenging to choose the treatment plan for patient #3 and patient #4 because the MiniPDX models showed resistance to all types of chemotherapeutic and targeted drug candidates. The patient #3 was treated with apatinib, and docetaxel combined with apatinib was used for patient #4. Both patients and their families were informed that the treatment effects might not be sound (Figure 2).

### 3.3. Side Effect Predictions and Measurements

**Figure 3:**
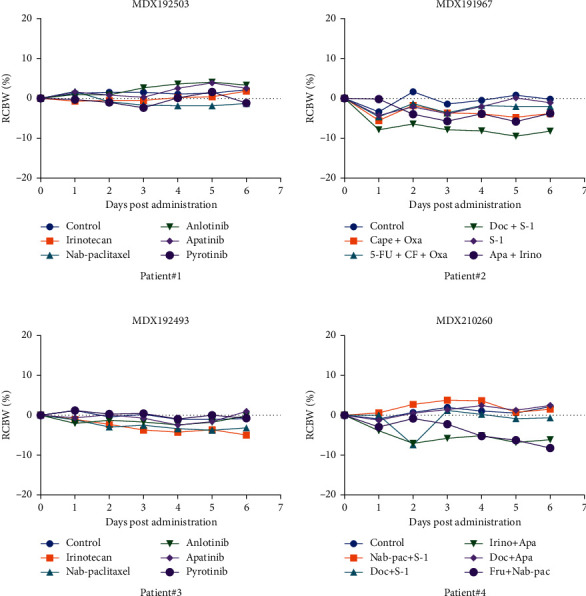
The loss of weight in mice during the 7-day drug treatment. RCBW% = (BWi − BW0)/BW0*∗*100%; BWi represents the body weight of the mice on day 1, while BW0 represents the body weight of mice when the MiniPDX model was first established. Abbreviations for chemotherapy: Nab-pac (nab-paclitaxel); Cape (capecitabine); Oxa (oxaliplatin); Irino (irinotecan); Apa (apatinib); doc (docetaxel); Fru (fruquintinib); 5-FU (5-fluorouracil); CF (calcium folinate).

Patients with advanced gastric cancer often have malnutrition and poor physical status. Therefore, plans chosen for each patient should consider not only the efficacy, but also the adverse reactions and toxicity. MiniPDX is the model that can predict not only a curative effect but also the toxicity. It is possible that the regimen would cause serious side effects if the mouse suffered a loss in body weight that exceeded 15% during the 7-day treatment. Alternatives were considered when the inhibitory rate was similar. The degree of weight loss for each mouse model is shown in Figure 3. We found that the nude mice paired with patients #1, patient #2, patient #3, and patient #4 did not show significant weight loss. Afterward, the patients did not show significant toxic and side effects after adopting the selective treatment plan. Mild side effects, according to the CTCAEv5.0 standards, are described in Table 3.

### 3.4. Treatment Outcomes

**Figure 4:**
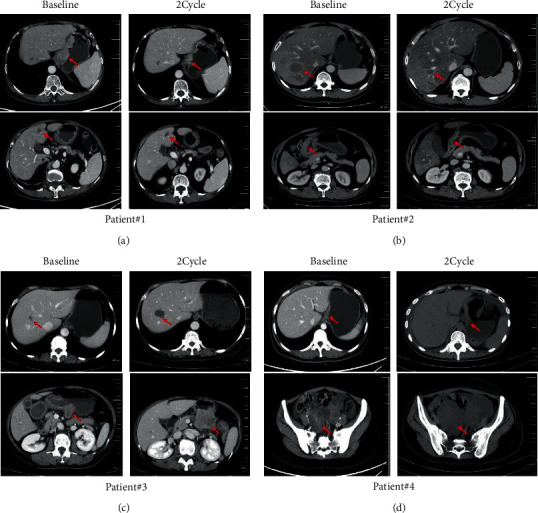
The clinical efficacy of drugs used in the patients based on the drug sensitivity results of the MiniPDX model. The red arrow indicates the location of the target lesion. CT scans were performed at the baseline and subsequent treatment cycles. Radiographic evidence of the four patients who achieved PR (patient #1, patient #2) or PD (patient #3, patient #4) after treatment was shown.

All 4 patients used relatively sensitive regimens based on the predicted results of the MiniPDX models. Following treatment, patient #1 and patient #2 reached partial response (PR) according to the Response Evaluation Criteria in Solid Tumors (RECIST) Version1.1 guidelines. However, patient #3 and patient #4 showed disease progression (Figure 4). The detailed treatment plans that were developed after drug selection are shown in Table 3. Specifically, patient #1 and patient #4 received sintilimab and camrelizumab, respectively, in the first-line treatment. Although the clinical data of immunotherapy in crossline treatment are few, the combination of immunotherapy with selected drugs was administered due to previous treatments with donated drugs.

## 4. Discussion

Patients with advanced gastric cancer have a short survival time and a poor overall prognosis. The median overall survival of patients with advanced disease (locally advanced or metastatic) was 10–12 months. [23]. Although various gastric cancer guidelines provide us with standardized diagnoses and treatment plans, due to individual differences and tumor heterogeneity [24], a certain plan is effective for one patient but ineffective in another. Therefore, a more precise plan to provide individualized treatment for each patient is urgently needed.

With the advent of the increasing number of targeted drugs and new chemotherapeutics, choosing individualized drugs for patients has become a challenge for clinicians. The PDX model could conserve the tumor microenvironment of the primary tumor. Compared to the previous cell-derived xenograft (CDX), PDX reserves the pathophysiology, histology, and phenotypic characteristics of primary tumors. The drug sensitivity test has a high consistency with clinical application, which is crucial in precise tumor treatment [25, 26]. Coclinical trials were run in parallel with human clinical trials in real time, and mouse trials using PDX models established from participants of clinical trials to evaluate drug response. This method is recognized as a model for personalized treatment or precision medicine [27, 28]. The PDX model used in coclinical trials is also called as “avatar” or “mirror” model. Some studies have reported that the patients and their PDX models had a high agreement rate of drug response [29, 30], indicating that these models can function as “mirror” models for donor patients. In addition, a PDX model can be treated not only with the same drug used in the donor patients but also with other drugs or a novel drug combination. The PDX model, in this case, an “avatar” model, can predict both the development of resistance to first-line therapy and the response to second-line therapy before these events are observed in the donor patient [31].

The advantage of the MiniPDX model is that we can quickly obtain the results of drug sensitivity experiments that are highly consistent with the PDX test results. Therefore, we can quickly adjust new treatment plans for patients based on the results of drug sensitivity test.

The drug sensitivity experiment technology of the MiniPDX model has been validated in the clinical research of colorectal cancer, hepatocellular carcinoma, gallbladder cancer, ovarian cancer, duodenal cancer, nonsmall cell lung cancer, small cell lung cancer, and other cancer types [19–21, 32–36].

In Li's research of metastatic colorectal cancer, the researchers used three kinase inhibitors (afatinib, gefitinib, and regorafenib) in 31 MiniPDX drug sensitivity testing. The study revealed that the primary tumor and metastases showed a different sensitivity to the same drug even in the same patient [32].

In Zhan's study of gallbladder cancer, the MiniPDX model was established using freshly resected primary lesions from 12 patients with gallbladder to examine the sensitivity of five of the most commonly used chemotherapeutic agents, namely, gemcitabine, oxaliplatin, 5-fluorouracil, nanoparticle albumin-bound (nab) paclitaxel, and irinotecan. The results were used to guide the selection of chemotherapeutic agents for adjunct treatment after the surgery. The Kaplan–Meier method was used to compare the overall survival (OS) and disease-free survival (DFS) of 12 patients in the test group and 45 patients in the control group who received conventional chemotherapy with gemcitabine and oxaliplatin. The result showed that patients in the MiniPDX-guided chemotherapy group had significantly longer median OS (18.6 months; 95% CI 15.9–21.3 months) than patients in the conventional chemotherapy group (13.9 months; 95% CI = 11.7–16.2 months) (*P*=0.030; HR = 3.18; 95% CI = 1.47–6.91). Patients in the MiniPDX-guided chemotherapy group also had significantly longer median DFS (17.6 months; 95% CI = 14.5–20.6 months) than patients in the conventional chemotherapy group (12.0 months; 95% CI = 9.7–14.4 months) (*P*=0.014; HR = 3.37; 95% CI = 1.67–6.79) [19].

The above studies have demonstrated that MiniPDX can perform drug sensitivity testing for different treatment options on different cancers, and patients can benefit from this technology. However, there was no study on gastric cancer using the MiniPDX model.

There were difficulties in the study. The first one is the location of the tissue biopsy. Some patients have liver metastases, and some have pelvic metastases. After the previous treatment, some of the primary tumors increased, and some of the metastases increased. Because of the difficulty of biopsy from metastases, we only collected tissues of primary gastric cancer. This may not be able to fully reflect the status of all tumors in the patients. The second one is how to evaluate immunotherapy. Immunotherapy has been approved for both the first-line and the third-line treatments of advanced gastric cancer. More and more patients choose a combination of chemotherapy and immunotherapy in the first-line treatment. There are no clinical research data for the maintenance and crossline treatment of immunotherapy in advanced gastric cancer. The MiniPDX model mice are immune system deficient, so only chemotherapy drugs and antiangiogenesis targeted drugs can be chosen as candidates. We often used combined immunotherapy on the basis of chemotherapy. However, whether such a combination is the best choice requires more clinical studies to confirm. The third challenge is how to discuss treatment options with patients. These four patients chose different treatment schemes based on the results of the drug sensitivity experiment of the MiniPDX model. Two patients were evaluated for efficacy as PR, and the other two patients were evaluated for efficacy as PD, which was consistent with the results of the MiniPDX model. Previous studies have shown that if the tumor suppression rate exceeds 55%, the chosen option is most likely to be ineffective. This brought us a lot of confusion. Shall we tell patients to give up treatment? Or continue to have a try? Is palliative treatment a better choice at this time? In addition, due to the limited research funding, our study was limited by a small sample size and a lack of the control group with patients received conventional chemotherapy. At the same time, we also failed to follow up with those 4 subjects to obtain further data such as OS and PFS. It is hoped that funding problems can be solved in our future research. A large randomized controlled clinical trial of MiniPDX technology applied to the drug sensitivity testing for advanced gastric cancer will be carried out.

Although the MiniPDX model has many advantages, it inevitably has some limitations. (1) The MiniPDX model is nonrenewable, requiring a continuous supply of fresh tumor tissue. (2) The usage and cycle of drugs in mice are different from those in humans. When the same drug is used in different cycles (for example, weekly albumin-bound paclitaxel vs. albumin-bound paclitaxel every 3 weeks), the MiniPDX model cannot simulate the actual administration and effect, because the drugs in the MiniPDX model are all used within 7 days. And, (3) the isolation of tumor tissue into single-cell suspension destroys the structure between cells, which may have a certain impact on the tumor microenvironment. Despite these shortcomings, the MiniPDX model still has outstanding advantages in the existing preclinical research models due to its short time-consuming, low cost, and ability to predict the drug treatment efficacy of patients. Especially when it is complementary to other platforms, the MiniPDX model can become a new platform in precision treatment research.

## 5. Conclusions

Our study demonstrated that the MiniPDX is a fast and effective screening model for antitumor drugs. It has particular significance for guiding the choice of drugs for patients with advanced gastric cancer. Recently, the integration of high-throughput sequencing and various indirect in vivo models has contribution to the rapid development of precision therapy. In the future, new and more efficient and comprehensive drug screening models need to be developed to help clinicians for rational use of drugs.

## Figures and Tables

**Table 1:** Baseline characteristics of patients.

Number	Age (y)	Gender	Tumor staging	Pathological types	Lauren classification; HER2 status	Metastasis sites	Previous treatment	MiniPDX models' chemotherapy regimen and doses.
#1	70	Female	IV	Adenocarcinoma	Intestinal type; HER2 positive	Liver lesions	1st line: sintilimab/trastuzumab/XELOX (8 cycles)	1, Irinotecan: 50 mg/kg, IP, day 1 and day 5;
								2, Nab-paclitaxel: 20 mg/kg, IV, day 1–day 5;
								3, Anlotinib: 3 mg/kg, PO, day 1–day 7;
								4, Apatinib: 100 mg/kg, PO, day 1–day 7;
								5, Pyrotinib: 10 mg/kg, PO, day 1–day 7;

#2	66	Male	IV	Adenocarcinoma	Intestinal type; HER2 positive	Liver lesions	1st line: SOX (4 cycles) + trastuzumab/SOX (3 cycles); maintenance treatment: trastuzumab + S-1	1, Oxaliplatin + capecitabine: (Oxa)5 mg/kg, IP, day 1+ (Cape), 400 mg/kg, PO, day 1–day 7;
								2, Oxaliplatin + CF + 5-FU: (Oxa)5 mg/kg, IP, day 1 + CF, 50 mg/kg, IP, day 1 + 5-FU, 25 mg/kg, IP, day 1–day 5;
								3, Docetaxel + S-1: (Doc)20 mg/kg, IP, day 1 and day 5 + S-1, 10 mg/kg, PO, day 1–day 5;
								4, S-1: S-1, 10 mg/kg, PO, day 1–day 5;
								5, Irinotecan + apatinib: (Iri)50 mg/kg, IP, day 1 and day 5+(Apa) 100 mg/kg, PO, day 1–day 7;

#3	67	Female	IV	Adenocarcinoma	Intestinal type; HER2 positive	Liver lesions	1st line: trastuzumab/XELOX (6 cycles)	1, Irinotecan: 50 mg/kg, IP, day 1 and day 5;
								2, Nab-paclitaxel: 20 mg/kg, IV, day 1–day 5;
								3, Anlotinib: 3 mg/kg, PO, day 1–day 7;
								4, Apatinib: 100 mg/kg, PO, day 1–day 7;
								5, Pyrotinib: 10 mg/kg, PO, day 1–day 7;

#4	34	Female	IV	Signet ring cell carcinoma	Diffuse type; HER2 negative	Pelvic cavity	1st line: camrelizumab/apatinib/SOX (3 cycles);	1, Nab-paclitaxel + S-1: nab-paclitaxel 20 mg/kg, IV, day 1–day 5 + S-1, 10 mg/kg, PO, day 1–day 5;
								2, Docetaxel + S-1: (Doc)20 mg/kg, IP, day 1 and day 5 + S-1, 10 mg/kg, PO, day 1–day 5;
								3, Irinotecan + apatinib: (Iri)50 mg/kg, IP, day 1 and day 5+(Apa)100 mg/kg, PO, day 1–day 7;
								4, Docetaxel + apatinib: (Doc): 20 mg/kg, IP, day 1 and day 5+(Apa)100 mg/kg, PO, day 1-day;
								5, Fruquintinib + nab-paclitaxel: (Fru)20 mg/kg, PO, day 1–day 7+(Pac)20 mg/kg, IV, day 1–day 5

Notes: Tumors were staged according to NCCN guidelines for gastric cancer (2021 V1). Metastasis sites were defined by the location of the main metastatic lesions.

**Table 2:** MiniPDX drug sensitivity results of the 4 patients.

Number	Chemotherapy regimen	Inhibition rate (1-T/C%)	Weight loss of mice >15%	Source of cancer cells
Patient #1	5, Irinotecan	68	—	Gastroscopic biopsy of gastric cancer
	6, Nab-paclitaxel	47	—	
	7, Anlotinib	46	—	
	8, Apatinib	52	—	
	9, Pyrotinib	55	—	

Patient #2	5, Oxaliplatin + capecitabine	20	—	Gastroscopic biopsy of gastric cancer
	6, Oxaliplatin + CF + 5-FU	16	—	
	7, Docetaxel + S-1	6	—	
	8, S-1	23	—	
	9, Apatinib + irinotecan	28	—	
Patient #3	5, Irinotecan	80	—	Gastroscopic biopsy of gastric cancer
	6, Nab-paclitaxel	>100%	—	
	7, Anlotinib	>100%	—	
	8, Apatinib	81	—	
	5, Pyrotinib	>100%	—	

Patient #4	5, Nab-paclitaxel + S-1	>100%	—	Gastroscopic biopsy of gastric cancer
	6, Docetaxel + S-1	>100%	—	
	7, Irinotecan + apatinib	>100%	—	
	8, Docetaxel + apatinib	66	—	
	9, Fruquintinib + nab-paclitaxel	71	—	

1, Notes: Inhibition rate was calculated by 1-T/C% (T/C% = treatment group proliferation rate/control group proliferation rate%). Each regimen was used on MiniPDX models with the same doses. Detailed doses for the MiniPDX models—irinotecan: 50 mg/kg, IP, day 1 and day 5; nab-paclitaxel, 20 mg/kg, IV, day 1–day 5; anlotinib, 3 mg/kg, PO, day 1–day 7; apatinib: 100 mg/kg, PO, day 1–day 7; pyrotinib, 10 mg/kg, PO, day 1–day 7; (oxaliplatin + capecitabine): oxaliplatin (Oxa), 5 mg/kg, IP, day 1 + capecitabine (Cape), 400 mg/kg, PO, day 1–day 7; (oxaliplatin + CF+5-FU): oxaliplatin, 5 mg/kg, IP, day 1 + CF, 50 mg/kg, IP, day 1 + 5-FU, 25 mg/kg, IP, day 1–day 5; (docetaxel + S-1): docetaxel (Doc): 20 mg/kg, IP, day 1 and day 5 + S-1, 10 mg/kg, PO, day 1–day 5; S-1: S-1, 10 mg/kg, PO, day 1–day 5; (irinotecan + apatinib): 50 mg/kg, IP, day 1 and day 5+ 100 mg/kg, PO, day 1–day 7; (nab-paclitaxel + S-1): nab-paclitaxel 20 mg/kg, IV, day 1–day 5 + S-1, 10 mg/kg, PO, day 1–day 5; (docetaxel + apatinib): docetaxel: 20 mg/kg, IP, day 1 and day 5 + apatinib: 100 mg/kg, PO, day 1–day 7; (fruquintinib + nab-paclitaxel): fruquintinib (Fru): 20 mg/kg, PO, day 1–day 7 + 20 mg/kg, IV, day 1–day 5.

**Table 3:** Treatment after enrollment, clinical response, and side effects.

Patient number	Treatment regimen after drug selection	Clinical outcome	Side effects
#1	Sintilimab: 200 mg d1, nab-paclitaxel: 180 mg d1, 8, q21d*∗*2 cycles	PR	Leukopenia (grade II)
			Thrombocytopenia (grade II)
			Alopecia (grade I)

#2	Docetaxel: 60 mg d1, 8, S-1: 60 mg PO bid d1-14, q21d*∗*2 cycles	PR	Alopecia (grade I)
			Leukopenia (grade I)

#3	Apatinib: 250 mg PO qd*∗*2 cycles	PD	Nausea (grade II)
			Anemia (grade II)

#4	Camrelizumab: 200 mg d1 q14 d, docetaxel: 60 mg d1, 8, apatinib: 250 mg PO qd*∗*1 cycle, q21d	PD	Nausea (grade II)
			Vomiting (grade II)
			Alopecia (grade I)

Notes: All drugs were used intravenously if not mentioned otherwise. Side effects were graded according to the CTCAEv5.0 standards.

## Data Availability

No data were used to support this study.
